# The association of Toll-like receptor 4 gene polymorphisms with the development of emphysema in Japanese subjects: a case control study

**DOI:** 10.1186/1756-0500-5-36

**Published:** 2012-01-18

**Authors:** Michiko Ito, Masayuki Hanaoka, Yunden Droma, Nobumitsu Kobayashi, Masanori Yasuo, Yoshiaki Kitaguchi, Toshimichi Horiuchi, Kayoko Ikegawa, Yoshihiko Katsuyama, Keishi Kubo, Masao Ota

**Affiliations:** 1 First Department of Medicine, Shinshu University School of Medicine, 3-1-1 Asahi, Matsumoto, Japan; 2 Department of Pharmacy, Shinshu University Hospital, 3-1-1 Asahi, Matsumoto, Japan; 3 Department of Legal Medicine, Shinshu University School of Medicine, 3-1-1 Asahi, Matsumoto, Japan

## Abstract

**Background:**

The principal role of Toll-like receptor 4 (TLR4) is the induction of immune responses to lipopolysaccharides. Previously, mice deficient in the *TLR4 *gene exhibited up-regulation of the NADPH oxidase system in the lungs. This resulted in increased oxidant generation and elastolytic activity, which led to pulmonary emphysema. It was suggested that TLR4 might maintain constitutive lung integrity by modulating oxidant generation. We investigated whether single nucleotide polymorphisms (SNPs) in the *TLR4 *gene were associated with the emphysema phenotype in Japanese subjects with chronic obstructive pulmonary disease (COPD).

**Results:**

Seven SNPs in the *TLR4 *gene (*rs10759930*, *rs1927914*, *rs12377632*, *rs2149356, rs11536889*, *rs7037117*, and *rs7045953*) were genotyped with allelic discrimination assays. The frequencies of SNPs were compared between 106 patients with the emphysema phenotype of COPD and 137 healthy smokers. We found that the positivity of the individuals with the major G allele of *rs11536889 *was significantly less in the emphysema group than the control group (*p *= 0.019). The frequencies of the minor C allele and the distribution of the CC genotype as well as the frequency of the major haplotype that carried the minor C allele of *rs11536889 *were all significantly higher in the emphysema group than the control group (*p *= 0.0083, 0.019, and 0.004, respectively). Furthermore, the strength of the association of the CC genotype with the emphysema phenotype was in an odds ratio of 2.60 with 95% confidence intervals from 1.17 to 5.78. However, these significances were not apparent after adjust for age and smoking history by logistic regression. No associations were observed between the *rs11536889 *and the low attenuation area score, the forced expiratory volume, and the carbon monoxide diffusion capacity in the emphysema group.

**Conclusions:**

The minor C allele of the *rs11536889 *SNP in the *TLR4 *gene is likely associated with the risk of developing emphysema in the Japanese population.

## Background

Chronic obstructive pulmonary disease (COPD) is a major global health problem that causes 64 million patients with COPD worldwide in 2004 and more than 3 million deaths in 2005 [1]. COPD is predicted to become the third most common cause of death and the fifth most common cause of disability in the world by 2020 [2]. The inflammation, extracellular matrix synthesis, oxidative stress and apoptosis are the major pathophysiological pathways in the mechanisms of COPD [3]. Recently, an animal model of autoimmune emphysema showed that CD4+ cell-dependent mechanisms were sufficient to trigger the development of emphysema, suggesting that alveolar septal cell destruction might result from immune mechanism [4]. The development of COPD is known to be influenced by multiple genetic factors [5]. A coding variant in surfactant protein B (SFTPB Thr131Ile) and the (GT)_31 _allele of the heme oxygenase (HMOX1) promoter short tandem repeat were evidenced to be associated with COPD in both the family-based study and case-control study [6]. In addition, TNF-α-308-1 and TNF-α-308-2 alleles, IL-13 promoter polymorphisms, metalloproteinase (TIMP)-2 polymorphisms, and β_2_-adrenoceptor Gly16 polymorphism were proved to be significantly associated with the presence of smoking-related COPD [7]. We also demonstrated that the transforming growth factor beta 1 gene polymorphisms were associated with emphysema phenotype of COPD in Japanese [8]. Recently, genome-wide association study identified bicaudal D homolog 1 (BICD1) as a susceptibility gene for emphysema [9]. However, few evidences have shown that genes involved with innate immune system are associated with emphysema or COPD.

The principal role of Toll-like receptors (TLRs) is the induction of immune responses. TLRs activate both innate and adaptive immune responses; they regulate the immediate response to pathogens and antigen presentation to the adaptive system [10,11]. Among the TLR family members, TLR4 is activated by bacterial lipopolysaccharides (LPS) [12]. LPS is a glycolipid component of Gram-negative bacteria cell walls; it is present in airborne particles, like tobacco smoke [13]. Recent evidence has demonstrated that mice deficient in the *TLR4 *gene developed pulmonary emphysema [14]. In that study, TLR4 knock out mice exhibited upregulation of a novel NADPH oxidase system in lungs and endothelial cells; this resulted in increased oxidant generation and elastolytic activity. However, various mediators of inflammation, including IL-1β, TNF-α, IL-6, IL-13, IFN-γ, and VEGF, were not differentially expressed. It is thought that TLR4 might maintain constitutive lung integrity by modulating oxidant generation. In another study, when TLR4 transgenic mice were exposed to hyperoxia, reduced apoptosis was observed in alveolar type I and II epithelial cells and alveolar macrophages. In addition, antiapoptotic molecules, like heme oxigenase-1, were up-regulated [15]. Those results suggested that TLR4 may function to protect against the development of emphysema through defending the oxidative stress and apoptosis.

The *TLR4 *gene is located on chromosome 9q32-33; it spans approximately 13 kb and contains three exons that encode a 222-amino acid protein. Single nucleotide polymorphisms (SNPs) in the *TLR4 *gene have been reported to be associated with endotoxin hyporesponsiveness and gram-negative infections [16-18]. *TLR4 *SNPs have been shown to affect the risks of various inflammatory diseases, including atherosclerosis [19], Crohn's disease [20], rheumatoid arthritis [21], and prostate cancer [22,23]. Studies on the association of *TLR4 *SNPs with COPD showed that the Asp299Gly (*rs4986790*) and Thr399Ile (*rs4986791*) polymorphisms of *TLR4 *gene were strongly associated with Caucasian patients [24-26]. However, these two functional SNPs were absent in the Japanese population according to the HapMap Project. At present, no *TLR4 *SNPs have yet been reported with the emphysema phenotype of COPD in Japanese subjects. In this case-control study, we investigated the association between *TLR4 *SNPs and COPD in Japanese subjects, with a focus on the emphysema phenotype.

## Results

### Characteristics of study subjects

Table 1 shows the characteristics and spirometric data of the study subjects. We selected 106 patients (102 males and 4 females) with the emphysema phenotype from 268 patients with COPD and 137 controls (133 males and 4 females) from healthy smokers for health screening in our afflicted hospitals. The gender ratio was matched between the case and control groups (*p *= 0.711), however, the age and smoking history were significantly higher in patients with emphysema compared to controls (*p *< 0.001). The FEV_1_/FVC and %FEV_1 _were significantly lower in patients with emphysema compared to control smokers (*p *< 0.001). Table 2 shows the information about the severity of the airflow limitation determined by GOLD classification and the total LAA score determined by Goddard's method in the emphysema group.

**Table 1:** Study subject characteristics and baseline spirometry data*

	Emphysema	Controls
Number of subjects	106	137
Male/Female	102/4	133/4
Age (years)	71.3 ± 6.8†	63.2 ± 10.0
Smoking history (pack-years)	63.3 ± 28.1†	39.6 ± 18.7
FEV_1 _(liter)	1.47 ± 0.66†	2.64 ± 0.51
FEV_1 _(% pred)	54.7 ± 22.8†	88.8 ± 13.1
FEV_1_/FVC (%)	48.4 ± 11.2†	80.3 ± 5.7

*Data are expressed as mean ± SD

*Abbreviations: FEV*_1 _forced expiratory volume in 1 second; % *pred *percent of the predicted value; *FVC *forced vital capacity

†*p *< 0.001 versus controls by the Mann-Whitney U test

**Table 2:** The severity of airflow limitation and total LAA scores in patients with emphysema phenotype

GOLD stage *	Number of patients (%)
Stage I	18 (17.0%)
Stage II	42 (39.6%)
Stage III	28 (26.4%)
Stage IV	18 (17.0%)

**Total LAA score **^†^	**Patient number (%)**

7-12	46 (43.4%)
13-18	24 (22.6%)
19-24	36 (34.0%)

*Abbreviations: GOLD *global initiative for chronic obstructive lung disease; *LAA *low attenuation area

*determined by GOLD spirometric classification

^†^determined by Goddard's method; scores represent total LAAs detected in 6 lung images

### Genetic information of the study subjects

**Figure 1:**
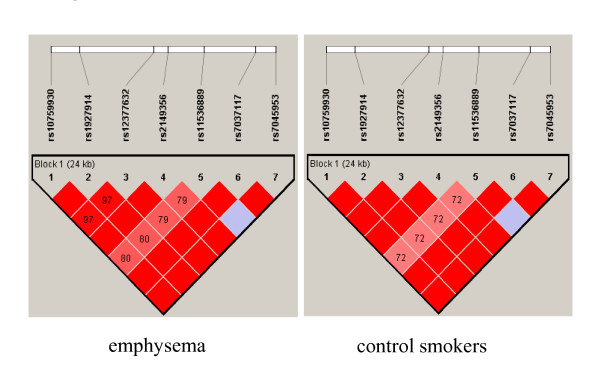
**Linkage disequilibrium (LD) plot of seven SNPs of the *TLR4 *gene**. LD plots were prepared for subjects with emphysema (left) and control smokers (right); *D'*_values that correspond to SNP pairs are expressed as percentages and shown within the respective squares. Higher *D' *values are indicated with a brighter red color. These seven SNPs constitute a haplotype block that spans 24 kb of the *TLR4 *gene.

The genotypic distributions of all seven SNPs in control smokers were in HWE (*p *> 0.2). The allelic positivities of the examined SNPs in the TLR4 gene are shown in Table 3. Compared to the control smokers, patients with emphysema had significantly lower positivity for the major G allele of *rs11536889 *(*p *= 0.019, odds ratio (OR) = 0.38, 95% confidence interval (CI) = 0.17-0.86). The other six SNPs did not show significant differences in allelic positivity rates between the two groups (Table 3). Further analysis regarding the *rs11536889 *revealed that the frequency of the minor C allele was significantly higher in the patients with emphysema phenotype than the health smokers (*p *= 0.0083), and the strength of the association of the C allele with emphysema phenotype was expressed by an OR of 1.69 with 95% CI from 1.14 to 2.50 (Table 4). Furthermore, the distribution of the CC genotype was significantly frequent in the emphysema group compared to that in the control group (*p *= 0.019), and the strength of the association of the CC genotype with emphysema phenotype was in an OR of 2.60 with 95% CI from 17 to 5.78 (Table 4). Figure 1 shows the pair-wise LD of the seven SNPs in patients with emphysema and control smokers. All SNP pairs showed high LD values, ranging from 0.79 to 1.00 in the cases and from 0.72 to 1.00 in control smokers. All SNP pairs showed so strong LD values that the Bonferroni method was not applied to adjust from multiple comparisons of the seven SNPs in order to avoid false negative results. The seven SNPs constituted a haplotype block in the *TLR4 *gene in both groups. Table 5 shows the nucleotide structures of the four most common haplotypes that contained the seven *TLR4 *SNPs and the observed frequencies in the two groups. The major haplotype, TACGCAA (with the minor C allele of *rs11536889*), occurred significantly more frequent in the case group (0.336) than the control group (0.219, *p *= 0.004), with an OR of 1.8 (95% CI: 1.21-2.70). The fourth most common haplotype, CGTTGAA (with the major G allele of *rs11536889*), was observed more frequently in the control smokers (0.165) than the patients with emphysema (0.100, *p *= 0.039), with an OR of 0.56 (95% CI: 0.33-0.97).

**Table 3:** Allelic positivities of single nucleotide polymorphisms (SNPs) in the *TLR4 *gene in Japanese subjects.

dbSNP*	Location Position	Allele	Emphysema (N = 106)	Controls (N = 137)	**O.R**.	*P*
					
			Allelic Positivity n (%)			
*rs10759930*	promoter 119,501,442	T C	92 (86.8) 62 (58.5)	111 (81.0) 83 (60.6)	1.54 0.92	0.229 0.741
*rs1927914*	promoter 119,504,546	A G	92 (86.8) 62 (58.5)	111 (81.0) 83 (60.6)	1.54 0.92	0.229 0.741
*rs12377632*	intron 2 119,512,551	C T	92 (86.8) 61 (57.5)	111 (81.0) 82 (59.9)	1.54 0.91	0.229 0.717
*rs2149356*	intron 2 119,514,020	G T	93 (87.7) 61 (57.5)	111 (81.0) 82 (59.9)	1.68 0.91	0.157 0.717
*rs11536889*	3'UTR 119,517,952	G C	88 (83.0) 58 (54.7)	127 (92.7) 58 (42.3)	0.38 1.65	0.019 0.055
*rs7037117*	3'UTR 119,523,484	A G	102 (96.2) 44 (41.6)	133 (97.1) 51 (37.2)	0.77 1.20	0.711 0.497
*rs7045953*	3'UTR 119,525,616	A G	106 (100) 18 (17.0)	137 (100) 21 (15.3)	- 1.13	- 0.728

Subjects included patients with emphysema and control smokers

*SNP designations are from the NCBI SNP database [29]. N = number of subjects. n = number of present alleles

*P *values were calculated with χ^2 ^tests of 2 × 2 contingency table

**Table 4:** Allelic frequency and genotypic distribution of the *rs11536889 *in the *TLR4 *gene in emphysema and control groups

*rs11536889*	Type	Emphysema (N = 106)	Controls (N = 137)	**O.R**.	*P*	*P^a^*
Allelic frequency, n (%)	G	136(64.2)	206 (75.2)	0.59	0.0083	
	C	76 (35.8)	68 (24.8)	1.69*		
Genotypic distribution, n (%)	GG	48 (45.3)	79 (57.7)	0.61	0.055	
	GC	40 (37.7)	48 (35.0)	1.12	0.664	0.034
	CC	18 (17.0)	10 (7.3)	2.60^‡^	0.019	

*N *number of subjects. *n *number of present alleles or genotypes correspondingly

*P *and *P^a ^*values were calculated with χ^2 ^tests of 2 × 2 and 3 × 2 contingency tables, respectively

*Odds Ratio = 1.69, 95% Confidence Intervals = 1.14-2.50

^‡^Odds Ratio = 2.60, 95% Confidence Intervals = 1.17-5.78

**Table 5:** The four most common haplotypes were comprised of the seven SNPs in the *TLR4 *gene.

SNPs	Haplotypes*			
	1	2	3	4
*rs10759930*	T	T	C	C
*rs1927914*	A	A	G	G
*rs12377632*	C	C	T	T
*rs2149356*	G	G	T	T
*rs11536889*	C	G	G	G
*rs7037117*	A	A	G	A
*rs7045953*	A	A	A	A
Freq. in emphysema	0.336	0.301	0.142	0.100
Freq. in control smokers	0.219	0.386	0.123	0.165
p^†^(emphysema vs. controls)	0.004^‡^	0.051	0.538	0.039^#^

The observed frequencies are shown for patients with the emphysema phenotype and control smokers

*Abbreviations: A *adenine; *C *cytosine; *Freq*. frequency; *G *guanine; *SNPs *single nucleotide polymorphisms; *T *thymine; *TLR4 *Toll-like receptor 4

*The designation of haplotype structure is given in Arabic numerals

^† ^By chi-square test

^‡^Odds Ratio = 1.80, 95% Confidence Intervals = 1.21- 2.70

^# ^Odds Ratio = 0.56, 95% Confidence Intervals = 0.33- 0.97

In addition, the %FEV_1_, %DLco, and LAA scores did not show any associations with the major or minor alleles of the seven SNPs in patients with the emphysema phenotype (data not shown).

## Discussion

The present study showed the minor C allele and the CC genotype of the *rs11536889 *SNP in the *TLR4 *gene were likely associated with the emphysema phenotype of COPD in Japanese subjects. The strength of the association of the CC genotype with emphysema phenotype was in an OR of 2.60. Moreover, the frequency of the major haplotype (Haplotype 1), which carried the minor C allele of *rs11536889*, was significantly higher in the emphysema group than in the controls. Another haplotype (Haplotype 4), which carried the major G allele of the *rs11536889*, was significantly higher in the in the controls than the emphysema group. However, we found that the clinical features of emphysema, such as %FEV_1_, %DLco and LAA scores were not significantly associated with the presence of *rs11536889 *in patients with emphysema.

TLRs are expressed in many cells, including airway epithelial cells, alveolar type II epithelial cells, alveolar macrophages, endothelium, fibroblasts, vascular smooth muscle cells, and T-cells [27]. TLR4s can recognize both LPS and a respiratory syncytial virus fusion protein [28]. The latter is a major respiratory pathogen in humans, infects the lower respiratory tract, and can exacerbate COPD. TLR4 can also be activated by proteins released from dead and dying cells, and tissue matrix breakdown products in the absence of infection, including high-mobility group protein 1 [29], surfactant protein A [30], fibronectin [31], fibrinogen [32], and hyaluronic acid oligosaccharides [33]. In smokers, stimulation with TLR4 agonists caused alveolar macrophages to reduce gene expression and secrete proinflammatory cytokines and chemokines [34]. Smokers and severe COPD patients displayed reduced *TLR4 *gene expression in the nasal epithelium. Consistent with that finding, a human epithelial cell line exposed to cigarette smoke extracts showed dose-dependent reductions in TLR4 mRNA and protein expression [35]. Those findings suggested that TLR4 might play a crucial role in the pathogenesis of COPD inflammation.

In the present study, the distribution of the *rs11536889 *CC genotype in control smokers was consistent with that reported for Japanese subjects in the HapMap project. In contrast, the CC genotype in the emphysema group was significantly higher than in the controls. The C allelic was also associated with emphysema phenotype. The *rs11536889 *C allele was previously demonstrated to be associated with moderate and severe periodontitis [36] and a high risk of gastric atrophy in Helicobacter pylori seropositive Japanese subjects [37]. However, the *rs11536889 *was not associated with autoimmune pancreatitis [38] or sarcoidosis-related uveitis [39] in Japanese subjects.

The *rs11536889 *SNP is located in the 3'-UTR of the *TLR4 *gene on chromosome 9q32-q33; it substitutes the ancestral guanine (G) with a mutated cytosine (C) at nucleotide position 3725. This SNP does not have any direct influence on the conformation of the TLR4 protein molecule, according to updated biogenetic data. However, it remains possible that this G/C mutation may have an effect on mRNA stability and transcription and/or translation efficiency; this might cause a dysfunction of the TLR4 molecule and interfere with the host immune system.

Two mutations in the *TLR4 *gene, Asp299Gly (*rs4986790*) and Thr399Ile (*rs4986791*), were reported to be highly associated with COPD in Caucasians [24-26], but these two functional SNPs were absent in the Japanese population according to the HapMap Project. At present, no other additional SNPs of the *TLR4 *gene have been reported to be associated with emphysema or COPD in Japanese or other populations worldwide.

The major limitation of the present case-control study was the relatively small sample sizes for both the case and control groups. The cases were strictly defined as the emphysema phenotype among the COPD patients by HRCT evaluation that was only applied to a part of the COPD patients due to several reasons of the patients. And the control group was recruited from the health centers where the apparent healthy people, mostly before retirement, voluntarily visit and take medical check-up once a year. As a result, the age and smoking history were significantly higher in the case group than the control group. When we analyzed the association of the *rs11536889 *SNPs with the emphysema phenotype, logistic regression was performed to adjust for age and smoking history as potential confounders. However, the significances in comparisons of the allelic frequency, genotype distribution and allele positivity of the *rs11536889 *between the emphysema and control smokers were not apparent. Logistic regression model allows adjustment for confounders with a representative random sample from the targeted study population assuming without bias [40], but that tends to systematically overestimate odds ratios when the sample size is less than about 500 [40]. Since our case group of emphysema phenotype was selected from the COPD population and the sample size was relatively small, we don't think logistic regression is suitable for the current adjustments. The odds ratio of 1.69 with 95% CI from 1.14 to 2.50 regarding the minor C allele and the odds ratio of 2.60 with 95% CI from 1.17 to 5.78 regarding the CC genotype of the *rs11536889 *provided a positive hint that the *rs11536889 *is important in the association with emphysema phenotype. With increasing sample size, the significances are supposed to approach the true population values [40]. We would expect replication studies for the association of the *rs11536889 *with emphysema in other Japanese groups. Further studies using appropriate animal models may verify the roles of *TLR4 rs11536889 *polymorphism in the pathogenesis of emphysema.

## Conclusions

This study demonstrated that the *rs11536889 *(+3725 G/C) SNP and the haplotype that carried the minor C allele of this SNP in the *TLR4 *gene were likely associated with the risk of developing the emphysema phenotype in the Japanese population.

## Methods

### Selection of cases

This study was approved by the Ethics Committee of Shinshu University. We obtained written informed consent from each case and control subject. Patients with COPD were recruited from the Department of Respiratory Medicine in Shinshu University Hospital (Matsumoto, Japan), and all participants were Japanese. COPD was diagnosed by smoking history, chronic respiratory symptoms (cough, sputum, breathlessness), and spirometric measurements that indicated an irreversible airflow limitation according to the Global Initiative for Chronic Obstructive Lung Disease (GOLD) [41]. Spirometry and carbon monoxide diffusion capacity (DLco) were measured with a pulmonary function testing system (Chestac-55 V, Chest Co. Ltd., Tokyo, Japan). Spirometry values were expressed as a percentage of the predicted value for forced expiratory volume in 1 second (%FEV_1_), which was based on equations formulated for Japanese. An airflow limitation was defined as a ratio of FEV_1 _to forced vital capacity (FVC) less than 70% (FEV_1_/FVC < 70%). The severity of the airflow limitation was determined according to spirometric classifications of GOLD [41], as follows: all stages showed FEV_1_/FVC < 70%; in addition, Stage I: %FEV_1 _≥ 80%; Stage II: 50% ≤ %FEV_1 _< 80%; Stage III: 30% ≤ %FEV_1 _< 50%; and Stage IV: %FEV_1 _< 30%.

Subjects with the following disorders were excluded from the study: late sequelae of pulmonary tuberculosis, diffuse panbronchiolitis, sinobronchitis, bronchiectasis, bronchiolitis obliterans due to autoimmune disease, and bronchial asthma.

The significant pathophysiological characteristic of COPD is airflow limitation that is caused by mixture phenotypes of small airway disease (obstructive bronchiolitis) and parenchymal destruction (emphysema), the relative contributions of which vary from person to person [42]. As a result, most patients with COPD have a combination of both phenotypes. The cases in the current case-control association study was strictly defined the patients with emphysema phenotype which was identified by high-resolution computed tomography (HRCT). A helical CT scanner (Hi Speed Advantage, Light Speed Ultra 16, or Light Speed VCT, GE Medical Systems, Milwaukee, WI) was used for HRCT scanning at full inspiration (total lung capacity level). We evaluated six slices, each 1.00-1.25 mm thick at three bilateral anatomic levels at full inspiration: the upper lung field (near the superior margin of the aortic arch), the middle lung field (at the level of the carina), and the lower lung field (at the level of the orifice of the inferior pulmonary veins). HRCT images were acquired with a window setting appropriate for the lungs (window level: -550 to -900 HU; width: 800 to 1500 HU). Low attenuation areas (LAA) were visually evaluated in each bilateral lung field according to the method of Goddard et al. [43] as follows: 0 = LAA < 5%; 1 = 5% ≤ LAA < 25%; 2 = 25% ≤ LAA < 50%; 3 = 50% ≤ LAA < 75%; 4 = 75% ≥ LAA. The total score was the sum of the scores for six slices (maximum score = 24). We defined the emphysema phenotype as a total score ≥ 7.

### Selection of controls

Control smokers were recruited from a population that underwent health screening at our affiliated hospitals (Misayama Hospital and Shinmachi Hospital, Japan). Control smokers were selected based on normal spirometric measurements. The selection criteria for controls were Japanese ethnicity, 50 years old or more, and former or current smoker. These criteria avoided ethnicity difference in this case-control association study, minimized the gaps of age and smoking history between the case and control groups, as well as gained large enough sample size to ensure statistical power for overcoming type I error.

### Genotyping

DNA was extracted from whole blood with the QuickGene-800 kit (FUJIFILM, Tokyo, Japan). Genomic DNA was prepared at concentrations of 5-15 ng/μl for the TaqMan SNP genotyping assay. Seven SNPs within the *TLR4 *gene were genotyped, including *rs10759930 *and *rs1927914 *in the 5' untranslated region (UTR); *rs12377632*, and *rs2149356 *in intron 2; *rs11536889*, *rs7037117*, and *rs7045953 *in the 3' UTR (Table 3). These SNPs included 5 kb of the predicted 5' UTR and 6 kb of the predicted 3' UTR, with minor allele frequencies > 5% according to the National Center for Biotechnology Information [44] SNP database. Genotyping the SNPs was performed with the SNP Genotyping Kit (Applied Biosystems, Tokyo, Japan). The polymerase chain reaction (PCR) was performed with a TaqMan Assay for Real-Time PCR (7500 Real Time PCR System; Applied Biosystems) according to the manufacturer's instructions.

### Statistical analysis

Continuous data are expressed as the mea n ± standard deviation (SD). For continuous variables, the differences between cases and controls were analyzed with the Mann-Whitney *U *test. We also tested the Hardy-Weinberg equilibrium (HWE) of each SNP among the controls. Allelic positivity was expressed in percentage. The positivity was defined as the frequency of individuals having one or two of the identical alleles in a given group. Differences in allele positivity between cases and controls were assessed with the Chi-square test (2 × 2 contingency table). The Haploview 3.32 program was used to compute pairwise linkage disequilibrium (LD) statistics [45]. The D' and r^2 ^values were plotted. LD blocks were defined according to the criteria of Gabriel et al [46]. Comparisons of haplotype frequencies between the cases and controls were performed with the Chi-square test. Phenotype-genotype associations were analyzed within the emphysema group by comparing the severity of emphysema (total LAA score), severity of airflow limitation (%FEV_1_), and deterioration of gas exchange (%DLco = a percentage of the predicted value for DLco) between patients with and those without either the major or minor allele of a SNP. The odds ratio (OR) and the approximate 95% confidence interval (CI) were calculated. Statistical significance was taken to be a p-value less than 0.05.

## Competing interests

The authors declare that they have no competing interests.

## Authors' contributions

MI drafted the manuscript. MI, MH, KK, and MO conceived and designed the study. MI, YK, and MO performed the statistical analysis and analyzed the data. MI, YD, and NK performed the genetic studies. MY, TH, and KI interpreted the data on lung function. All authors read and approved the final manuscript.
